# Midday and nadir salivary cortisol appear superior to cortisol awakening response in burnout assessment and monitoring

**DOI:** 10.1038/s41598-018-27386-1

**Published:** 2018-06-14

**Authors:** Alexander Pilger, Helmuth Haslacher, Bernhard M. Meyer, Alexandra Lackner, Selma Nassan-Agha, Sonja Nistler, Claudia Stangelmaier, Georg Endler, Andrea Mikulits, Ingrid Priemer, Franz Ratzinger, Elisabeth Ponocny-Seliger, Evelyne Wohlschläger-Krenn, Manuela Teufelhart, Heidemarie Täuber, Thomas M. Scherzer, Thomas Perkmann, Galateja Jordakieva, Lukas Pezawas, Robert Winker

**Affiliations:** 1 Department of Laboratory Medicine, Medical University of Vienna, Vienna, Austria; 2 Health and Prevention Center, Sanatorium Hera, Vienna, Austria; 3 Gruppenpraxis Labors.at, Vienna, Austria; 4 Empirical Research, Vienna, Austria; 5 Department of Physical Medicine, Rehabilitation and Occupational Medicine, Medical University of Vienna, Vienna, Austria; 6 Department of Psychiatry and Psychotherapy, Medical University of Vienna, Vienna, Austria

## Abstract

Burnout and work-related stress symptoms of anxiety disorder and depression cause prolonged work absenteeism and early retirement. Hence, reliable identification of patients under risk and monitoring of treatment success is highly warranted. We aimed to evaluate stress-specific biomarkers in a population-based, “real-world” cohort (burnouts: n = 40, healthy controls: n = 26), recruited at a preventive care ward, at baseline and after a four-month follow up, during which patients received medical and psychological treatment. At baseline, significantly higher levels of salivary cortisol were observed in the burnout group compared to the control group. This was even more pronounced in midday- (p < 0.001) and nadir samples (p < 0.001) than for total morning cortisol secretion (p < 0.01). The treatment program resulted in a significant reduction of stress, anxiety, and depression scores (all p < 0.001), with 60% of patients showing a clinically relevant improvement. This was accompanied by a ~30% drop in midday cortisol levels (p < 0.001), as well as a ~25% decrease in cortisol nadir (p < 0.05), although not directly correlating with score declines. Our data emphasize the potential usefulness of midday and nadir salivary cortisol as markers in the assessment and biomonitoring of burnout.

## Introduction

Prevalence rates of work-related mental health problems have dramatically increased over the last decade and are challenging national welfare systems^1^. Indeed, in 2013, 5.7% of the Austrian workforce considered psychological stress as their main work-related health concern^2^. Likewise, 4.9% reported to have suffered from depressive and anxiety symptoms (mostly women)^2^. Excessive work-related stress might culminate in burnout, a state of total exhaustion, which comprises of various physical, mental, behavioral and emotional symptoms in response to excessive job demands^3^. Although psychological burnout has already been described many years ago^4,5^, accurate physiological parameters to measure it have remained elusive. Since burnout represents a severe detraction in the qualified workforce at its most productive years, it is urgent to devise better ways to measure it in burnout candidates as well as in patients under treatment^6^.

Increased psychological stress was suggested to affected hypothalamus-pituitary-adrenal (HPA) axis regulation^7,8^ and, consequently, increase the risk for psychiatric illnesses such as major depression (MDD) and anxiety disorders. This association between work-related psychological stress and psychiatric disease is coined in the term burnout^3,9^, which does neither represent a novel psychiatric disease nor a causal relationship between specific occupational social interactions and disease onset. According to Freudenberger, who is regarded as the founder of the concept of burnout, the syndrome proceeds in twelve phases, which do not necessarily occur sequentially: 1) the obligation to prove oneself, 2) increased engagement, 3) neglect of own needs, 4) displacement of conflicts and desires, 5) reinterpretation of values, 6) denial of problems, 7) withdrawal from social environment, 8) change in behavior, 9) depersonalization, 10) inner void, 11) depression, and 12) total exhaustion^9^.

However, there is no doubt that psychiatric disorders are increasing in working patients, thereby leading to prolonged sick leaves (30–50%)^10^ and premature retirement (32.8%)^11^ in Austria and other welfare countries. Our cohort of municipal employees typically report “burnout symptoms” reflecting merely work-related stress symptoms of mild to moderate MDD and anxiety disorder within classical DSM-IV boundaries^12^. In contrast to psychiatric inpatients, these primary care patients are typically underdiagnosed and subsequently undertreated. Hence, a population-based model is required to provide information for initial screening and treatment to this often neglected subpopulation at early stages of disease^13^.

As mentioned above, reliable biomarkers for early diagnosis of burnout and monitoring of treatment success are still missing^14^. We recently identified a set of peripherally assessable biomarkers amongst professional orchestra musicians in a performance situation^15^.Those markers have been reported to exhibit immunological messenger or effector function and to act as proxy for psychological stress. Amongst them was cortisol, which has repeatedly been implicated in mechanistic models of major depression or anxiety disorders^16^. Briefly, psychological stress is thought to be processed by the hippocampus, which serves as a brain-environment interface and should optimally prepare the organism for environmental changes. On a hormone level, this was reflected by alterations in the release into the blood circulation of cortisol releasing hormone (CRH). Increased CRH levels impact on brain function resulting in adaptive behavior in healthy subjects or maladaptive behavior and psychopathological symptoms in patients^17^. Moreover, hormonal signaling across the HPA axis also leads to increases in cortisol levels during situations of acute psychological stress or a blunted response^18^; especially the cortisol awakening response (CAR), which is the rapid increase in cortisol levels across the first 30–45 min following morning awakening, represents a promising and convenient parameter that is associated with depressive symptoms and psychological stress^19^. Additionally, in our musician cohort we found associations between increased psychological stress levels and interleukin 6 (IL6), myeloperoxidase (MPO), and homocysteine (HCYS)^15^. All of these compounds can promote oxidative stress, which may trigger neuropsychiatric disorders by damaging brain structures^20^. In brief, IL6 is a mainly pro-inflammatory cytokine, which also responds to mental stress^21^ and is linked to mood disorders through the cytokine model of depression^22^. MPO is the main antimicrobial effector molecule of neutrophilic granulocytes^23^. When released into the surrounding tissue during host defense or in response to psychological stress^24^, its peroxidase activity promotes inflammation, and damages also endogenous structures via generation of reactive oxygen and nitrogen species^25^. HCYS is potentially toxic, especially when it occurs within the nervous system, and is generated by demethylation of methionine^26^. The substance is naturally eliminated by both folate and vitamin B_12_, however, accumulation of HCYS precedes vascular events^27^ and correlates with the presence of neuropsychiatric disorders^28,29^. Further support for the utility of these latter three parameters stems from independent studies showing that acute psychological stress leads to an increase of circulating IL6^30^, MPO^31^, and HCYS levels^32^.

Despite the abundance of reports highlighting the association between stress-related psychopathological symptoms and changes in blood parameters, there is still a debate on how such indicators^14^ could be utilized for diagnostic purposes. To improve disease management, we assessed cortisol day profiles, but also IL6, MPO, and HCYS plasma concentrations so as to complement clinical psychological measurements in a representative cohort of burnout patients during a four month period at our stress outpatient clinic. We hypothesized higher saliva cortisol levels in so called “burnout” patients prior to clinical response and relative to healthy controls. Moreover, in secondary hypotheses we expected peripheral markers of oxidative stress to be increased ahead of treatment in this patient group possibly useful as future disease marker.

## Methods

### Patient recruitment and Study design

**Figure 1:**
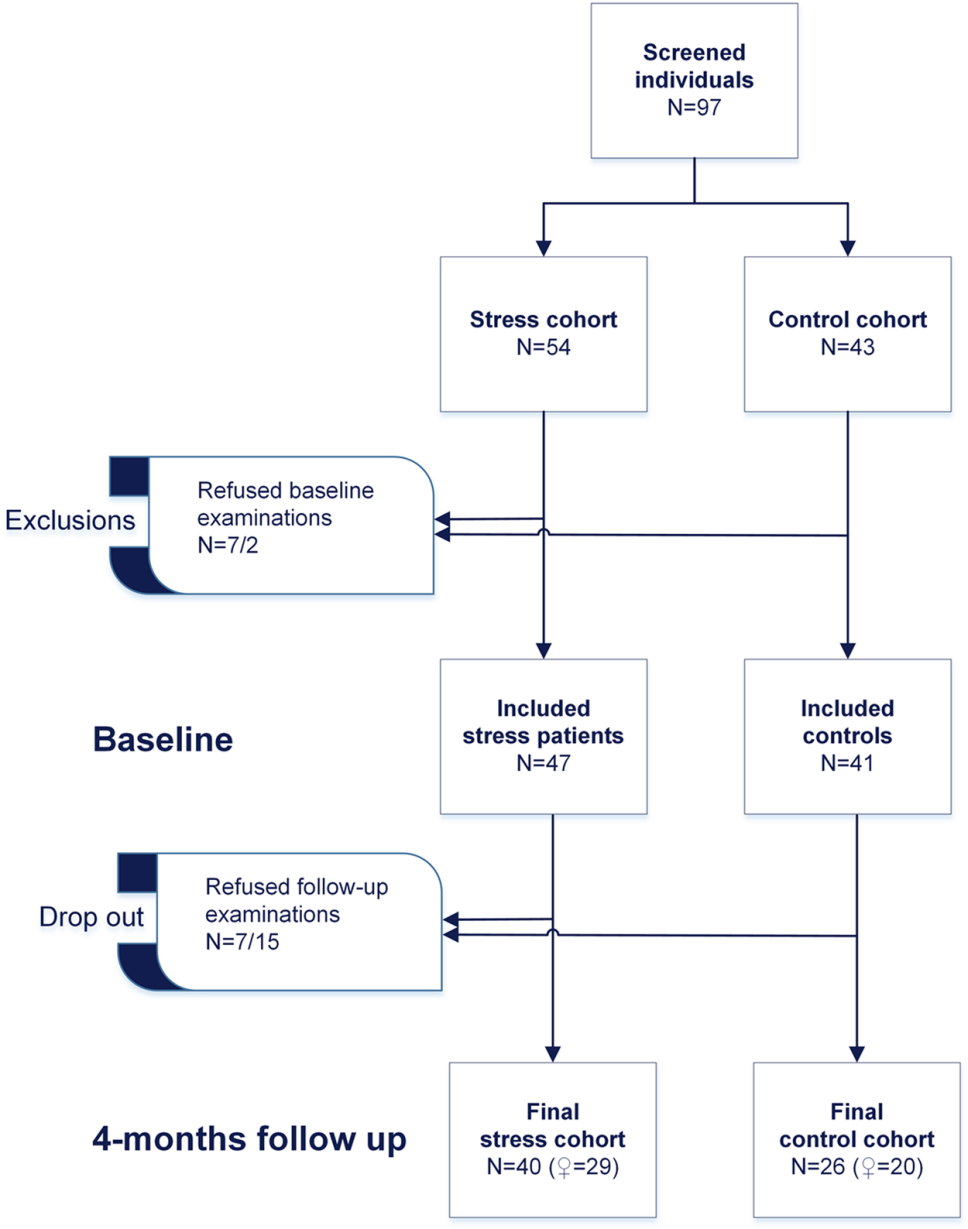
Study flowchart. ♀, females.

Outpatients of the Health and Prevention Center, which is the primary Healthcare Institution for municipal employees of the City of Vienna, were screened for depressive, anxiety, and unspecific work-related stress symptoms. Data collection was conducted between 09/2014 and 11/2016 utilizing a comprehensive psychosocial screening questionnaire (PSS) as being detailed below. Patients of both sexes were included beyond classical DSM-IV boundaries in line with the recently proposed Research Domain Criteria (RDoC) approach^12^. For this, the following inclusion criteria were applied: (1) ability to provide written informed consent, (2) commitment to fulfill the study protocol including baseline and follow-up visits, (3) age older than 18 yrs., and (4) exceeding cut-off scores in HADS depression, HADS anxiety (≥8), or a T-value of PSS stress sum-score ≥60. In contrast, (1) pregnancy, (2) non-compliance with the study protocol, or (3) a current treatment with glucocorticoids were defined as exclusion criteria. Clinical axis I main diagnoses were assessed according to DMS-IV by experienced and board-certified psychiatrists after study inclusion (see sample characteristics). All participants underwent a thorough medical exam at baseline, as well as pre- and posttreatment blood withdrawal, PSS assessments, and evaluation of saliva cortisol day profiles. The group of patients was provided an open four-month lasting treatment comprising intensive medical and psychological assistance (see below). In addition, a control cohort, which did not differ in terms of age and sex, without any concurrent psychiatric diagnosis was consecutively recruited at the Health and Prevention Center. Recruitment procedures are depicted in Fig. 1.

This study as well as all applicable amendments were approved by the Ethics Committee of the Medical University of Vienna (071/2009) and was performed in accordance with the Declaration of Helsinki and other relevant guidelines/regulations. All participants gave written informed consent prior to study participation.

### Psychosocial Screening Questionnaire

The PSS questionnaire covers frequently observed mental health issues optionally assessed by following published and validated subscales within a low-threshold preventive care service at the Health and Prevention Center, Sanatorium Hera. Following data were collected via paper/pencil or online (http://service.hera.co.at/psychoscreen/): Depression and anxiety items were derived from the Hospital-Anxiety and Depression Scale (HADS), whose 2-factorial structure is well established and both subscales can be considered highly reliable (retest reliability in the interval of two weeks > 0.8)^33^. Work-related stress was assessed based on the burn-out risk screening according to the 12-phase model of Freudenberger and North^9^. This screening instrument results in a validated factorial structure of twelve sub-scales corresponding to the phases and a sum score transformed in a T-value. It has proved to be highly consistent (Cronbach’s α = 0.937)^34^. Finally, lifestyle parameters were assessed including the Personal-Life-Style-Questionnaire (PLQ), the Fagerström nicotine dependency^35^ and the Audit-GMAT for alcohol dependency^36^.

Symptom severity (CGI-S) was assessed at each visit utilizing the Clinical Global Impressions (CGI) Scale by a board certified psychiatrist on a scale ranging from 1 (“normal”) to 7 (“among the most extremely ill patients”), with 2 as a cut-off for marginally ill patients^37^.

### Collection of samples

Participants were fully instructed regarding the preanalytical requirements of saliva collection and asked to adhere to the following procedure the day before baseline and follow-up examinations: At-home collection of salivary samples 1) immediately after awakening, as well as 2) 15 minutes, 3) 30 minutes and 4) 45 minutes after awakening, 5) between 11:30 a.m. and 12:30 p.m., and 6) directly before going to bed. They were not allowed breakfasting (incl. caffeinated or sugared drinks) or smoking during the post-awakening phase. To improve compliance with salivary cortisol sampling we specifically emphasized to the participants the importance of accurate sample collection and reporting their sampling times^38,39^. The protocol was explained in detail to participants, thereby focusing on what is meant by the ‘moment of awakening’. In addition, subjects were informed that analysis of post-awakening cortisol levels requires strict adherence to the sampling schedule, since delays of 10 to 15 min might already affect results^40^. To increase compliance, participants also kept a diary to record collection time points and activities during the day. Samples were stored at approximately 4 °C at the patients’ residences until they were transferred to the Department of Laboratory Medicine. Blood withdrawal took place before medical checkup and psychological testing. Sample logistics was accomplished in cooperation with the MedUni Wien Biobank facility (www.biobank.at).

### Laboratory analyses

Plasma concentrations of myeloperoxidase (MPO), interleukin 6 (IL6), homocysteine (HCYS), as well as salivary cortisol levels were measured at the Department for Laboratory Medicine, Medical University of Vienna, which, as the central facility for laboratory diagnostics at the General Hospital of Vienna, harbors a certified (ISO 9001:2008) and accredited (ISO 15189:2012, not applicable for myeloperoxidase) quality management system (www.kimcl.at).

In detail, MPO was quantified from EDTA-anticoagulated plasma by means of enzyme-linked immunosorbent assays (ELISA), using commercially available, IVD-certified MPO ELISA kits (Immundiagnostik AG, Bensheim, Germany), featuring 4–5% intra-assay and 12–15% inter-assay reproducibility. IL6 was measured from EDTA-anticoagulated whole-blood using commercially available IL6 electrochemiluminescent immunoassays (ECLIA) kits (Roche Diagnostics GmbH, Mannheim, Germany) on a cobas e602 module (Roche Diagnostics GmbH) with an intra-assay precision between 1.4 and 6.0% and an inter-assay reproducibility between 2.7 and 8.5%. In order to prevent *in-vitro* production of HCYS by erythrocytes, EDTA-anticoagulated whole blood was cooled after withdrawal and plasma was at the earliest possible time point separated from blood cells. HCYS was quantified by enzyme-cycling assays, using the oxidative capacity of homocysteine on cobas c701 modules (Roche Diagnostics GmbH). Cortisol was assessed from saliva collected in Salivette® cortisol saliva tubes (Sarstedt AG & Co., Nuembrecht, Germany) by means of ECLIA on cobas e602 modules (Roche Diagnostics GmbH).

### Treatment Period

The Health and Prevention Center, Sanatorium Hera, provides a collaborative^41^ and population-based care^13^ by a multidisciplinary team of mental health professionals including psychiatrists, psychologists and psychotherapists, and specialists from other medical fields, such as physiotherapy, sports science and occupational medicine. Collaborative management of outpatients was coordinated weekly. Individual treatment strategies were implemented for specific clinical conditions according to patient’s need and illness severity to maximize treatment adherence^42^ and to provide a cost-effective case management^13^. Treatment in face-to-face and group settings followed current S3 treatment guidelines of depression^43^ in terms of following treatment elements: patient education, pharmacological therapy, psychological and psychotherapeutic treatment including debriefing and stress coping, mindfulness based stress reduction/cognitive therapy (MBSR/MBCT), biofeedback, physical activation and relaxation techniques (e.g. progressive muscle relaxation, PMR).

### Statistics

Continuous data were presented as median (quartile 1 – quartile 3) or, if appropriate, as mean and 95% confidence interval of the mean. Continuous data were given as counts and percentages. Differences in continuous data between groups were assessed by non-parametric Mann-Withney U tests, differences in nominal data by cross-tabulation and Pearson’s χ² tests. Correlations between continuous variables were calculated according to Spearman and given as Spearman’s ρ. The time courses of continuous variables were evaluated by two-way ANOVA with repeated measurements design (SPSS general linear models), giving readings of a continuous quantity (dependent variable) at two levels of a within-subject factor, and a dichotomous characteristic (e.g. group assignment) as independent, between-subjects factor. Interactions between the within-subject factor and the between-subject factor indicate that the within-subject factor develops different between both time points in the categories of the between-subject factor (e.g. results of a biomarker decline between baseline and follow-up examinations in group A, whereas they stagnate or even rise in group B).

Areas under the cortisol curve (AUC) have been calculated from 4 subsequent measurements (time of awakening, 15, 30, and 45 minutes after awakening) according to Pruessner *et al*.^44^, and given as AUC_g_ and AUC_i_. In detail, AUC_g_ (area under the curve with respect to ground) represents an approximated integral of the total saliva cortisol concentration over the first 45 minutes after awakening and is given in µg × h/dl:

1 $$AU{C}_{g}=\,0.125\times (Cortiso{l}_{0\text{'}}+2\times Cortiso{l}_{15\text{'}}+2\times Cortiso{l}_{30\text{'}}+Cortiso{l}_{45\text{'}})$$

When subtracting the basal level measured at the time of awakening, integrated over the 0.75 hours of measurement, from the aforementioned AUC_g_, only the cortisol changes occurring due to the awakening response are taken into account. The resulting value is defined as AUC_i_ (area under the curve with respect to increment):

2 $$AU{C}_{i}=AU{C}_{g}-Cortiso{l}_{{0}^{\text{'}}}\times 0,\,75$$

Notably, the term “cortisol awakening response (CAR)” is exclusively reserved for measurements of the post-awakening dynamic, e.g. AUC_i_^45^. Results being below the analytical lower limit of quantification (LLOD) were set to their arithmetical expected value (E_(X)_) at LLOQ/2:

3 $${E}_{(x)}=\frac{0+LOD}{2}$$

Effect sizes were given with respect to the statistical tests applied: Mann-Whitney U test, r; Pearson’s χ² test, φ; two-way ANOVA with repeated measurements design, partial η². Multiple testing correction of p-values was conducted according to Benjamini and Hochberg, and adjusted p-values were given as p_BH_. p-values < 0.05 were considered statistically significant. All calculations were made in SPSS 23.0 (IBM, Armonk, USA) and MedCalc Statistical Software (MedCalc Software bvba, Ostend, Belgium), figures were mainly drawn using GraphPad Prism (GraphPad Software Inc., La Jolla, USA).

### Data availability

Since publication of raw data was not consented by the study participants, data will not be openly available. However, raw data sets can be requested from the corresponding author by interested researchers.

## Results

### Baseline characteristics

**Figure 2:**
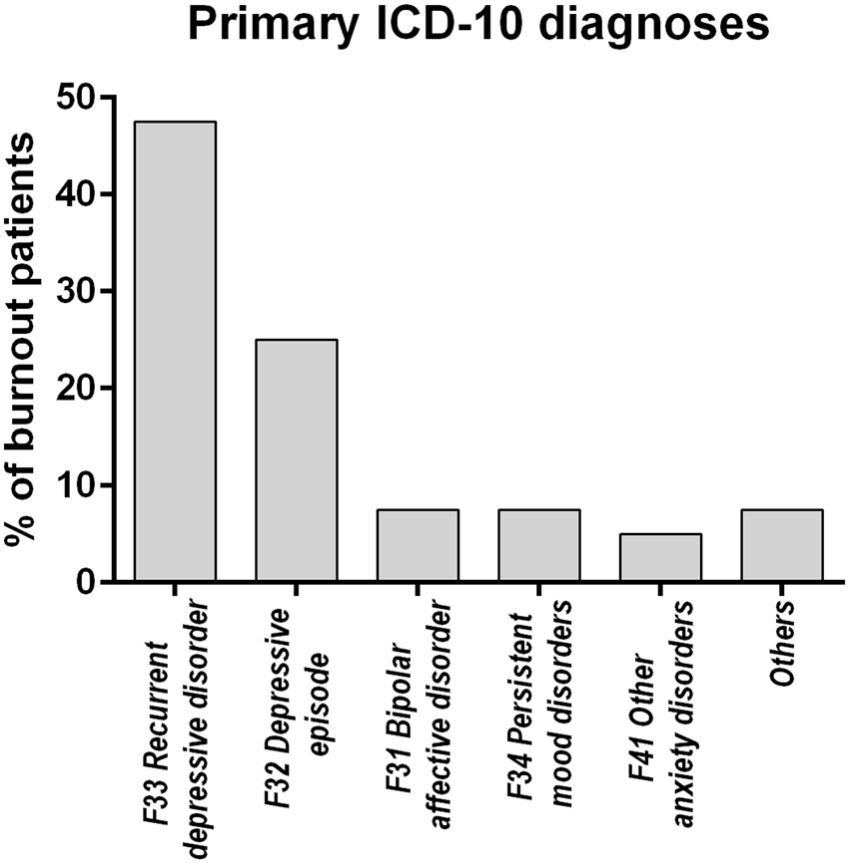
Primary ICD-10 diagnoses within burnout patients (N = 40).

Detailed baseline data are displayed in Table 1. 40 burnout patients (73% female; age 49 years [39–53]) and 26 participants of the control group (77%, 43 [39–53]) completed pre- and posttreatment assessments. Psychosocial screening values above cut-offs (HADS anxiety and depression subscales), as well as a clinical global impression ≥2 were mandatory for inclusion into the stress cohort. Primary ICD-10 diagnoses are given in Fig. 2; those were dominated by *Recurrent depressive disorder* (F33, 47.5%), followed by *Depressive episode* (F32, 25%), *Bipolar affective disorder* (F31, 7.5%), *Persistent mood disorders* (only Dysthymia, F34.1, 7.5%) and *Other anxiety disorders* (only Generalized anxiety disorder, F41.1, 5%). “Others” account for 7.5% and comprise of *Neurasthenia*, *Bulimia nervosa* and *Habit and impulse disorder* (each 2.5%). As intended, both groups did not significantly differ regarding age (p = 0.382, p_BH_ = n.s.) and sex (p = 0.688, p_BH_ = n.s.). Interestingly, burnout patients showed a higher body mass index (BMI = 26.1 [22.5–30.8] vs. 22.7 [21.4–24.6], p = 0.007, p_BH_ < 0.05). However, BMI did not interact significantly when included as a confounding variable (data not shown).

**Table 1:** Baseline characteristics of both, the stress cohort and the age- and sex-matched control group.

	Stress cohort	Control cohort	Effect size	p	p_BH_
Age	49 (39–53)	43 (32–52)	0.11	0.382	n.s.
Female sex	29 (73%)	20 (77%)	−0.049	0.688	n.s.
Smoker	9 (23%)	2 (8%)	0.194	0.115	n.s.
BMI [kg/m²]	26.1 (22.5–30.8)	22.7 (21.4–24.6)	0.33	**0**.**007**	<**0**.**05**
CGI-S	3 (3–4)	1 (1–1)	0.84	<**0**.**001**	<**0**.**001**
Questionnaires					
Burnout-risk screening	66.7 (58.0–73.4)	42.5 (40.8–47.0)	0.81	<**0**.**001**	<**0**.**001**
HADS anxiety	11.5 (8.3–14.0)	2.5 (2.0–4.0)	0.79	<**0**.**001**	<**0**.**001**
HADS depression	9.0 (6.3–14.8)	3.0 (1.0–4.0)	0.76	<**0**.**001**	<**0**.**001**
Blood markers					
HCYS [µmol/l]	11.0 (10.0–13.7)	12.1 (10.4–14.2)	0.17	0.169	n.s.
IL6 [pg/ml]	2.5 (0.9–3.4)	1.5 (0.8–2.6)	0.24	0.054	n.s.
MPO [ng/ml]	158 (108–203)	122 (100–189)	0.15	0.215	n.s.
Saliva cortisol day profile					
Awakening [µg/dl]	0.63 (0.41–0.75)	0.38 (0.26–0.46)	0.46	<**0**.**001**	<**0**.**001**
Awakening + 15′[µg/dl]	0.68 (0.43–0.80)	0.48 (0.31–0.62)	0.34	**0**.**005**	<**0**.**05**
Awakening + 30′ [µg/dl]	0.71 (0.47–0.93)	0.50 (0.42–0.64)	0.31	**0**.**012**	<**0**.**05**
Awakening + 45′ [µg/dl]	0.67 (0.50–0.75)	0.42 (0.27–0.66)	0.32	**0**.**009**	<**0**.**05**
AUC_g_ [µg × h/dl]	0.50 (0.37–0.62)	0.35 (0.26–0.46)	0.39	**0**.**002**	<**0**.**01**
AUC_i_ [µg × h/dl**]**	0.04 (−0.06–0.14)	0.06 (0.02–0.16)	0.11	0.379	n.s.
midday [µg/dl]	0.27 (0.18–0.35)	0.14 (0.06–0.20)	0.56	<**0**.**001**	<**0**.**001**
nadir [µg/dl]	0.18 (0.13–0.27)	0.05 (0.05–0.05)	0.65	<**0**.**001**	<**0**.**001**

Categorical data was compared by Pearson’s χ² tests, continuous data was compared by Mann-Whitney U tests. Effect sizes for Mann-Whitney U tests are given as r, for Pearson’s χ² tests as φ. Adjusted p-values (according to Benjamini and Hochberg) below 0.05 were considered statistical significant.

CGI-S, Clinical Global Impression Scale – Severity; HCYS, homocysteine; IL6, interleukin 6; MPO, plasma myeloperoxidase; AUC_g_, area under the curve with respect to ground; AUC_i_, cortisol awakening response – area under the curve with respect to increase; PSS, psychosocial screening^34^; HADS, Hospital anxiety and depression scale^61^.

### Baseline cortisol levels differ between burnout patients and controls

To validate the proposed stress biomarkers, baseline levels of blood- or saliva-borne molecules were measured in samples obtained from the 40 burnout patients, including cortisol, IL6, MPO, and HCYS. Marker levels were determined using commercially available immunoassays. Healthy volunteers (N = 26) were considered as controls. Results are summarized in Table 1.

Patients showed higher saliva cortisol concentrations for all awakening samples, with strongest effects for sample five and with the exception of sample three (30 minutes after awakening) and, accordingly, presented with a significantly higher AUC_g_ value (p = 0.002, p_BH_ < 0.01) when compared to the control group. However, differences in cortisol levels measured at later points in time (noon/evening) were even more pronounced, with patient levels failing to decrease during the day or at nighttime (both p/p_BH_ < 0.001).

When comparing blood-borne biomarkers between stress patients and controls, we could not detect any significant differences. Though, IL6 showed trend-wise elevated levels within stress patients (p = 0.054, p_BH_ = n.s.). Apart from that, we found an association between baseline IL6 and BMI among patients (ρ = 0.371, p = 0.019, p_BH_ < 0.05), but not in controls (p = 0.237, p_BH_ = n.s.). At follow up, this association was no longer detectable (ρ = 0.289, p = 0.070, p_BH_ = n.s.; controls: p = 0.840, p_BH_ = n.s.). Hence, the proposed parameters showed no discriminatory capabilities that could be used in identification of stress patients.

Based on these data, it would appear that for the use of cortisol as a marker of burnout, midday and evening cortisol levels were at least as efficient as total salivary cortisol after awakening. In a next step, it was examined whether the investigated parameters might also allow for longitudinal monitoring of psychological stress in response to treatment.

### Treatment of burnout patients corresponds to lower cortisol levels

**Figure 3:**
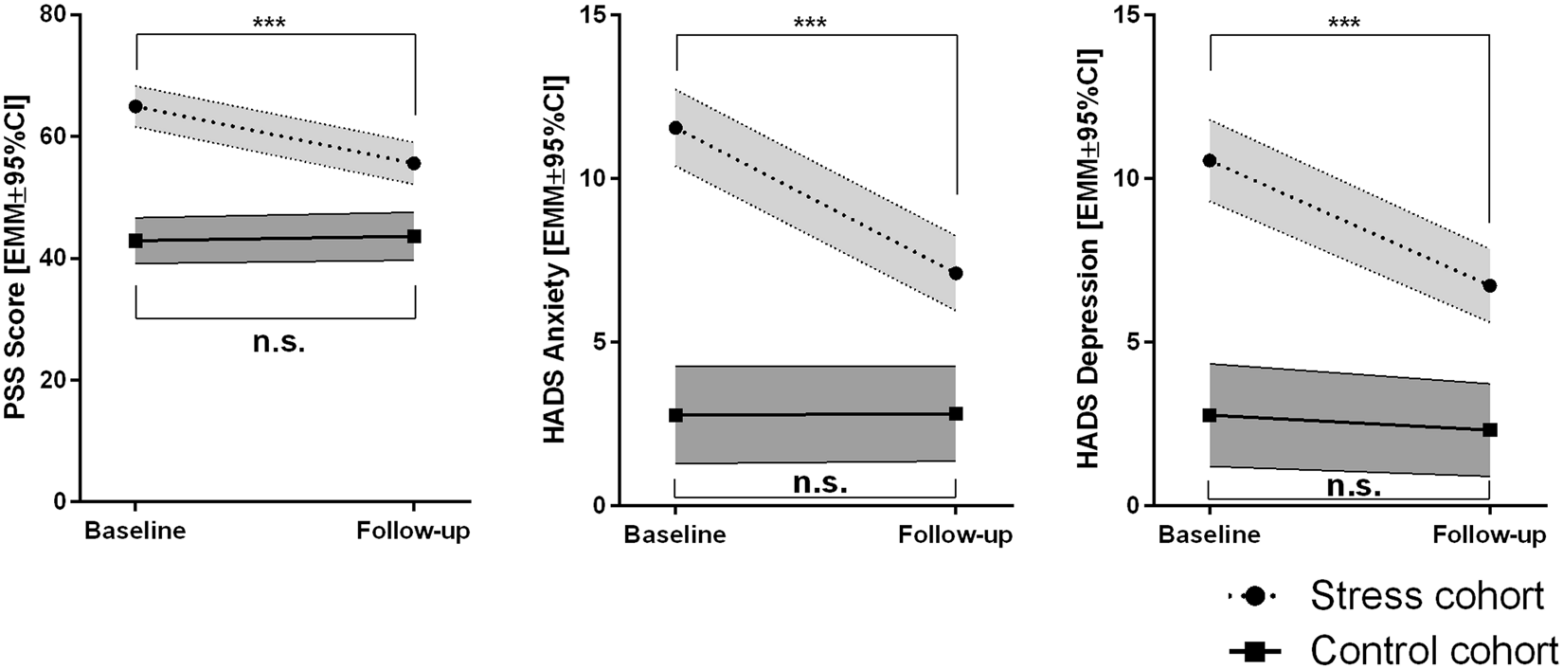
Temporal development of relevant psychosocial scores. Estimated marginal means are calculated by general linear models (two-way ANOVA with repeated measurements design) and p-values were adjusted according to Benjamini and Hochberg. EMM, estimated marginal mean; 95%CI, 95% confidence interval, PSS, psychosocial screening^34^; HADS, Hospital anxiety and depression scale^61^; n.s., not significant; ***p_BH_ < 0.001.

To determine whether cortisol measurement might be useful in monitoring therapy success in burnout patients, these were first assessed on a behavioral level. The aforementioned 40 burnout patients underwent a four month period of combined treatment, with a median of five (2–10) personal sessions, whereby the actual number of visits was very specifically oriented towards the individual patients’ needs and requirements. By filling out appropriate psychosocial screening questionnaires (PSS, HADS anxiety and depression scores), they were evaluated by trained psychological personnel. Healthy volunteers, which did not receive any neuro-psychological treatment in this period of time, were assessed in analogy to patients. General linear models with repeated measurements design were used to arrive at statistical significance. Results are given in Fig. 3 and Table 2.

**Table 2:** Test statistics for general linear models (two-way ANOVA with repeated measurements design) assessing differences in relevant scores between baseline and follow-up examinations (main effects score), differences in neuropsychological scores between stress cohort and control group (main effect group), and, whether temporal developments of scores vary between groups (interaction group × score).

	Main effect score	Main effect group	Interaction group × score
PSS Score	F = 9.192 df_1_ = 1, df_2_ = 50 partial η² = 0.155 **p = 0**.**004**, **p**_**BH**_** < 0**.**01**	F = 68.621 df_1_ = 1, df_2_ = 50 partial η² = 0.578 **p < 0**.**001**, **p**_**BH**_** < 0**.**001**	F = 12.536 df_1_ = 1, df_2_ = 50 partial η² = 0.200 **p = 0**.**001**, **p**_**BH**_** < 0**.**01**
HADS Anxiety	F = 18.025 df_1_ = 1, df_2_ = 64 partial η² = 0.220 **p < 0**.**001**, **p**_**BH**_** < 0**.**001**	F = 75.613 df_1_ = 1, df_2_ = 64 partial η² = 0.542 **p < 0**.**001**, **p**_**BH**_** < 0**.**001**	F = 18.659 df_1_ = 1, df_2_ = 64 partial η² = 0.226 **p < 0**.**001**, **p**_**BH**_** < 0**.**001**
HADS Depression	F = 19.258 df_1_ = 1, df_2_ = 64 partial η² = 0.231 **p < 0**.**001**, **p**_**BH**_** < 0**.**001**	F = 58.942 df_1_ = 1, df_2_ = 64 partial η² = 0.479 **p < 0**.**001**, **p**_**BH**_** < 0**.**001**	F = 11.857 df_1_ = 1, df_2_ = 64 partial η² = 0.156 **p = 0**.**001**, **p**_**BH**_** < 0**.**01**

Effect sizes are given as partial η². P-values given as p_BH_ have been adjusted according to Benjamini and Hochberg. Df, degrees of freedom; PSS, psychosocial screening^34^; HADS, Hospital anxiety and depression scale^61^.

Healthy volunteers maintained a stable score in the PSS questionnaire (PSS: p = 0.735, p_BH_ = n.s.; HADS anxiety: p = 0.962, p_BH_ = n.s.; HADS depression: p = 0.546, p_BH_ = n.s.). In contrast, burnout patients yielded a mean reduction of 9.3 ± 10.2 points in the stress score, 4.5 ± 4.1 in the anxiety score, 3.8 ± 3.9 and in the depression scale (all p/p_BH_ < 0.001). Likewise, the median CGI-S which was 3 (3–4) at baseline, significantly decreased along treatment (−1 [−1–0]; p/p_BH_ < 0.001). Sixty percent of patients where treatment responders with more than one third of patients showing full remission at their final visit (CGI-S = 1: 5% [n = 2]; CGI-S = 2: 33% [n = 13]).

The results indicated that biopsychosocial treatment was beneficial to the burnout patients. Subsequently, it was evaluated whether this improvement would also be seen in reductions in biomarker levels over the treatment period.

**Figure 4:**
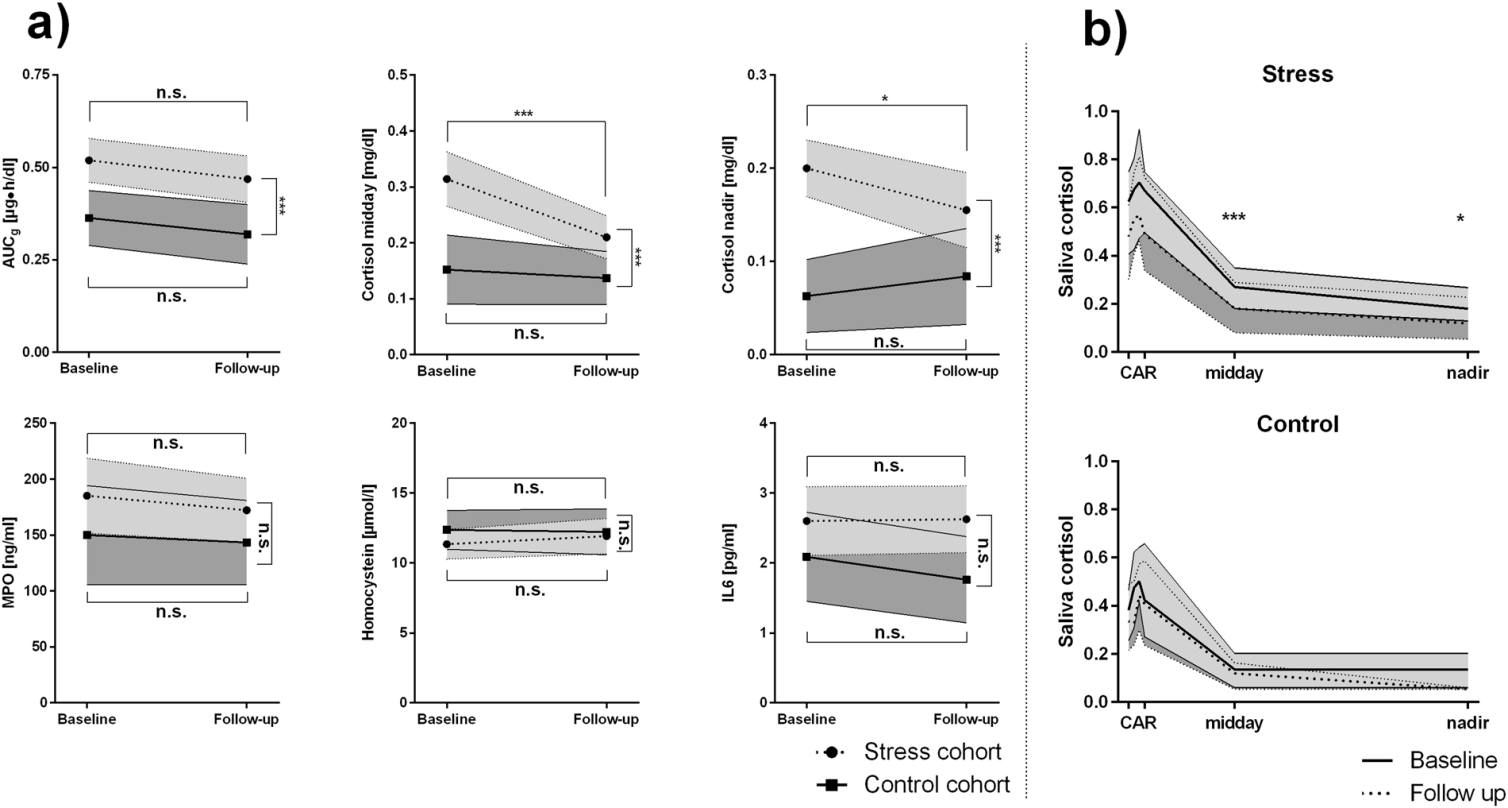
(**a**) Temporal development of biomarkers. Estimated marginal means are calculated by general linear models (two-way ANOVA with repeated measurements design) and p-values were adjusted according to Benjamini and Hochberg. Data is given as estimated marginal mean and 95% confidence interval, and confidence intervals from stress patients are light grey shaded, whereas confidence intervals from controls are dark grey shaded. (**b**) Variation in cortisol day profiles at baseline (light grey) and before follow-up examinations (dark grey) within the stress (top) and the control cohort (bottom). AUC_g_, area under the curve with respect to ground; AUC_i_, cortisol awakening response – area under the curve with respect to increase; MPO, plasma myeloperoxidase; IL6, interleukin 6; n.s., not significant; ***p_BH_ < 0.001; *p_BH_ < 0.05.

Therefore, the same patient group was asked to provide a saliva day profile and blood samples, once at the onset of therapy and once at its termination, and these were subjected to the aforementioned analyses. The control group was similarly assessed. Test statistics are presented in Table 3, temporal developments of biomarkers are visually depicted in Fig. 4.

**Table 3:** Test statistics for general linear models (two-way ANOVA with repeated measurements design) assessing differences in biomarkers between baseline and follow-up examinations (main effects score), differences in biomarker levels between stress cohort and control group (main effect group), and, whether temporal developments of biomarker levels vary between groups (interaction group × biomarker).

	Main effect biomarker	Main effect group	Interaction group × biomarker
AUC_g_	F = 3.243 df_1_ = 1, df_2_ = 62 partial η² = 0.050 p = 0.077, p_BH_ = n.s.	F = 14.846 df_1_ = 1, df_2_ = 62 partial η² = 0.193 **p < 0**.**001**, **p**_**BH**_** < 0**.**001**	F = 0.025 df_1_ = 1, df_2_ = 62 partial η² < 0.001 p = 0.875, p_BH_ = n.s.
AUC_i_	F = 0.032 df_1_ = 1, df_2_ = 62 partial η² = 0.001 p = 0.858, p_BH_ = n.s.	F = 0.014 df_1_ = 1, df_2_ = 62 partial η² < 0.001 p = 0.905, p_BH_ = n.s.	F = 1.210 df_1_ = 1, df_2_ = 62 partial η² = 0.019 p = 0.276, p_BH_ = n.s.
Cortisol midday	F = 10.608 df_1_ = 1, df_2_ = 62 partial η² = 0.146 **p = 0**.**002**, **p**_**BH**_** < 0**.**01**	F = 16.158 df_1_ = 1, df_2_ = 62 partial η² = 0.207 **p < 0**.**001**, **p**_**BH**_** < 0**.**001**	F = 5.999 df_1_ = 1, df_2_ = 62 partial η² = 0.088 **p = 0**.**017**, **p**_**BH**_** < 0**.**01**
Cortisol nadir	F = 0.636 df_1_ = 1, df_2_ = 63 partial η² = 0.010 p = 0.428, p_BH_ = n.s.	F = 19.847 df_1_ = 1, df_2_ = 63 partial η² = 0.0240 **p < 0**.**001**, **p**_**BH**_** < 0**.**001**	F = 4.434 df_1_ = 1, df_2_ = 63 partial η² = 0.066 **p = 0**.**039**, p_BH_ = n.s.
MPO	F = 0.787 df_1_ = 1, df_2_ = 62 partial η² = 0.013 p = 0.378, p_BH_ = n.s.	F = 2.012 df_1_ = 1, df_2_ = 62 partial η² = 0.031 p = 0.161, p_BH_ = n.s.	F = 0.082 df_1_ = 1, df_2_ = 62 partial η² = 0.001 p = 0.775, p_BH_ = n.s.
HCYS	F = 0.263 df_1_ = 1, df_2_ = 61 partial η² = 0.004 p = 0.610, p_BH_ = n.s.	F = 0.632 df_1_ = 1, df_2_ = 61 partial η² = 0.010 p = 0.430, p_BH_ = n.s.	F = 0.758 df_1_ = 1, df_2_ = 61 partial η² = 0.012 p = 0.387, p_BH_ = n.s.
IL6	F = 0.650 df_1_ = 1, df_2_ = 63 partial η² = 0.010 p = 0.423, p_BH_ = n.s.	F = 4.122 df_1_ = 1, df_2_ = 63 partial η² = 0.061 **p = 0**.**047**, p_BH_ = n.s.	F = 0.879 df_1_ = 1, df_2_ = 63 partial η² = 0.014 p = 0.352, p_BH_ = n.s.

Effect sizes are given as partial η². P-values given as p_BH_ have been adjusted according to Benjamini and Hochberg. Df, degrees of freedom; AUC_g_, area under the curve with respect to ground; AUC_i_, cortisol awakening response – area under the curve with respect to increase; MPO, plasma myeloperoxidase; HCYS, homocysteine; IL6, interleukin 6.

**Figure 5:**
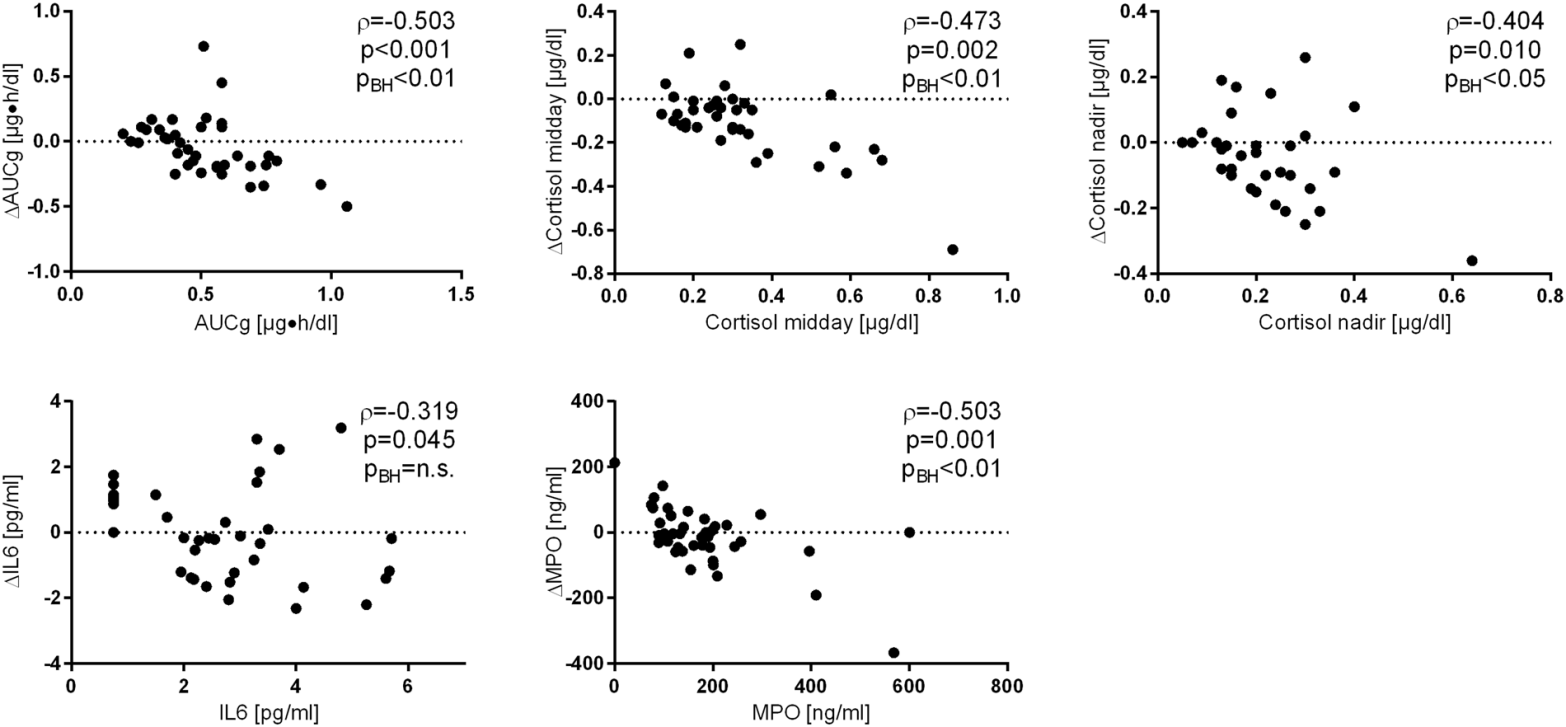
Interdependence between baseline laboratory results (X-axes) and therapeutic response (Y-axes) among patients of the intervention group. Correlation coefficients are calculated according to Spearman. AUC_g_, area under the curve with respect to ground, AUC_i_; cortisol awakening response – area under the curve with respect to increase; MPO, plasma myeloperoxidase; IL6, interleukin 6.

Of the assessed stress associated biomarkers, only the temporal developments of midday cortisol and saliva cortisol nadir showed a significant statistical interaction with the group variable (although no longer statistically significant after p-value adjustment), indicating that both values decreased, as expected, in the burnout group, but not in controls. In detail, investigated patients presented with a midday cortisol decline between baseline and follow-up of −0.10 ± 0.14 µg/dl (p/p_BH_ < 0.001) and also with a slightly less pronounced evening cortisol drop of −0.05 ± 0.14 (p = 0.022, p_BH_ < 0.05). Interestingly, biomarker dynamics during the therapeutic phase highly depended on baseline levels, as higher baseline concentrations correlated with a less pronounced decrease at follow-up (see Fig. 5) for most parameters (all p/p_BH_ < 0.001).

However, changes of biomarkers after the treatment phase did in neither case correlate with any improvement in one of the stress, mood or anxiety-related questionnaires (all p/p_BH_ > 0.05). The significance of the utility of saliva cortisol, sampled conveniently at noon or at the evenings, to diagnose and monitor burnout is discussed.

## Discussion

Burnout syndrome is gaining medical importance in the industrialized world, whereby people at their height of productivity might not be able to work any further, thereby meaning not only a major social-economic loss^46^, but additionally being exposed to serious suffering. Despite the ever increasing incidence of burnout^11^, reliable biomarkers to help with diagnosis or treatment monitoring are still missing^14^. Thus, the objective of this study was to determine whether cortisol day profiles, IL6, MPO, and HCYS plasma concentrations could be utilized as biomarkers in such patients with respect to clinical course and treatment outcome. To our knowledge, we showed here for the first time in a prospective setting that cortisol levels sampled at midday and in the evenings were not inferior to the more complex cortisol awakening response in distinguishing burnout patients and healthy controls.

In the present study, we measured saliva cortisol day profiles as well as plasma IL6, MPO and HCYS at baseline and in response to a four months treatment. At the clinical level, stress relief during the course of treatment was subjectively reported by anxiety, depression, and burnout-risk questionnaires, and objectively assessed by CGI ratings (Table 1). At the follow up visit the majority of patients (60%) showed a significant clinically improvement (indicated by a drop in the CGI score) that allowed patients to resume their work highlighting the socioeconomic value associated with such treatment programs. The results of this study demonstrated that the night time, midday (11:30–12:30) and, to a lesser extent, also morning cortisol concentrations were useful biomarkers for work-related stress symptoms when compared between patients and healthy controls, and within patients during recovery from major depression and anxiety disorders. Whereas blood-borne biomarkers did not exhibit any discriminatory or predictive usefulness, saliva cortisol levels presented with considerable diagnostic accuracy. Interestingly, cortisol samples taken either at midday or night time appeared to be superior to the more complex assessment of the cortisol awakening response. To our knowledge, this is the first study that prospectively monitored biomarkers of inflammation and oxidative stress during multiprofessional collaborative treatment^41^ of work-related stress patients recruited beyond classical DSM-IV limitations according to RDoC principles^12^. The applied design was superior compared to cross-sectional studies regarding causal inference on longitudinal processes^47^. Hence, it is tempting to speculate that the observed symptom alleviation might be linked to the reduction of cortisol levels following therapy.

Various parameters of cortisol secretion have been applied in studies on psychiatric disorders. Regarding baseline levels, there are many studies reporting no difference in afternoon cortisol concentrations between controls and patients with current major depression or anxiety disorders^16^. By contrast, older adults suffering from depression were found to display significantly higher levels of basal cortisol than healthy controls during all phases of diurnal cycle but particularly during the evening and night hours^48^. Similar to these results, we have observed that, compared to controls, our burnout group had higher cortisol levels during the day, with the most significant value in the evening. Notably, degrees of depression, anxiety and stress were well reflected in AUC_g_ values and even more pronounced in the nadir levels of cortisol. Treatment of the patients caused a decrease in midday and nadir cortisol only, whereas the concentrations of morning cortisol appeared to be unaffected.

The cortisol awakening response is the most prominent component of the diurnal cortisol profile investigated in psychosocial and mental health studies^38^. Deviations from a regular CAR profile are assumed to indicate maladaptive neuroendocrine processes. Meta-analysis of the AUC_i_ showed positive associations between the magnitude of the AUC_i_ and occupational stress and stressful life-events^49^. By contrast, there was a tendency for negative correlations between AUC_i_ and fatigue, exhaustion, or burnout, the latter representing a symptom cluster with quite inconsistent results^50–54^. However, the present study neither identified an altered level of AUC_i_ in the burnout group nor any statistically significant association between AUC_i_ and symptom scores. This is in line with previous studies showing no relationship between burnout questionnaire and CAR^51^, and no impact of depression, hopelessness and negative affect on CAR^49^. The fact that we found equal AUC_i_ values in patients and controls whilst the burnout group exhibited an overall elevation of the cortisol profile may be explained by the well-known uncoupling of CAR from basal circadian cortisol secretion^38^. A similar effect has been reported when assessing differences in CAR in relation to severity of burnout^52^. Patients with low burnout displayed lower basal levels of cortisol than patients with moderate burnout whereas the relative increase in cortisol over the first 30 min after morning awakening appeared to be unchanged. Taken together, these results point to some limitation of morning AUC_i_ for comparisons of cortisol patterns in psychiatric disorders.

In our study, midday and nadir cortisol showed the most pronounced correlations with HADS anxiety score. In addition, a significant correlation between AUC_g_ and anxiety score was found, whereas no association between AUC_i_ and degree of anxiety could be observed. A possible explanation could be that the more complex assessment of morning response curves produces a higher pre-analytical variability^38^. Indeed, measures of dispersion are slightly higher in AUC_g_ (Standard error of the mean, SEM = 0.024) when compared to midday (SEM = 0.020) or nadir cortisol (SEM = 0.014). Above that, cortisol hypersecretion is clearly associated with depressive behavior^55^. Especially rumination, which is an aggravating symptom of burnout and depression^56^, was shown to delay the cortisol decline during the day^57^. Since especially nadir concentrations of healthy individuals were hardly measurable and presented with very low variability, the difference to burnout patients might become more obvious. Moreover, our results support the findings of Merswolken *et al*.^58^, who demonstrated on the one hand a significant correlation between anxiety and AUC_g_, and on the other hand only a trend towards an association between anxiety and AUC_i_ in CHD patients. A recent report by Hakamata *et al*.^59^ provides a possible mechanistic model for the relationship between cortisol and anxiety by underlining the importance of the strength of amygdala-hippocampus functional connectivity.

IL6, MPO, HCYS plasma concentrations did not change within subjects during recovery from stress, and did not differ between patients and controls. Even though IL6 was trend-wise higher in the patient cohort, these measures might, according to our findings, not be useful as biomarkers to monitor stress relief in patients.

This study comes with two limitations, which, however, did not markedly affect quality: First, the sample size appears prima facie to be relatively small compared to the number of covariates. However, correlations between changes in biomarkers and psychosocial variables presented with very low effect sizes (ρ < 0.25), which might probably not be diagnostically relevant. Therefore, the statistical detection of those relationships would have no clinical value. The differences in sample size between control and intervention group can be considered irrelevant as well, since relevant parameters (e.g. age, sex) are equally distributed in both groups. Taking this together, our sample size can be considered as sufficient for testing clinically relevant interdependencies. Indeed, inspired by previous clinical studies^60^, we preferred a naturalistic and population-based setting to ensure realistic conditions with higher external validity. The second limitation concerns saliva pre-analytics. Of course, subjects were informed about the necessity of accurate sample collection at home. However, temporal accuracy cannot be completely assured, since collections were not monitored by study personnel. To enhance compliance, subjects were required to document the exact time points in a day protocol.

In conclusion, the present results encompass that high levels of stress, depression, and anxiety are reflected by increased concentrations of salivary cortisol, especially just before going to bed at night (nadir). Although patients responded on a clinical level, neither of the assessed blood parameters showed to be a predictor of clinical treatment outcome. Hence, our study suggests that measurement of salivary cortisol might be a useful objective adjunct in addition to clinical diagnostics, and, to a lesser extent, as objective marker for disease course. Notably, single cortisol values from saliva collected at noon or bedtime exhibited considerably better diagnostic quality than more expensive and error-prone sequences, as e.g. the cortisol awakening response, a variable which has led to inconsistent results in the biomonitoring of burnout^14^.
